# Effects of Climate, Soil, Topography and Disturbance on Liana Prevalence

**DOI:** 10.1002/ece3.70374

**Published:** 2024-10-10

**Authors:** Emma J. Mackintosh, Catherine E. Waite, Francis E. Putz, Marion Pfeifer, Chengrong Chen, Zhongming Lan, Sophie Brennan, Andrew R. Marshall

**Affiliations:** ^1^ Forest Research Institute University of the Sunshine Coast Sippy Downs Queensland Australia; ^2^ Department of Zoology University of Cambridge Cambridge UK; ^3^ Modelling, Evidence and Policy Research Group, School of Natural and Environmental Sciences Newcastle University Newcastle upon Tyne UK; ^4^ School of Environment and Science Griffith University Brisbane Queensland Australia; ^5^ School of Environmental and Conservation Sciences Murdoch University Perth Western Australia Australia

**Keywords:** Australian wet tropics, environmental drivers, rainforest, rattans, recovery, vines

## Abstract

Lianas (woody vines and climbing monocots) are increasing in abundance in many tropical forests with uncertain consequences for forest functioning and recovery following disturbances. At a global scale, these increases are likely driven by disturbances and climate change. Yet, our understanding of the environmental variables that drive liana prevalence at regional scales is incomplete and geographically biased towards Latin America. To address this gap, we present a comprehensive study evaluating the combined effects of climate, soil, disturbance and topography on liana prevalence in the Australian Wet Tropics. We established 31 20 × 20 m vegetation plots along an elevation gradient in low disturbance (canopy closure ≥ 75%) and high disturbance (canopy closure ≤ 25%) forest stands. In these plots, all tree and liana (defined as all woody dicot vines and climbing monocots, i.e., rattans) stems ≥ 1 cm DBH were measured and environmental data were collected on climate, soil and topography. Generalised linear models were used with multi‐model averaging to quantify the relative effects of the environmental variables on measures of liana prevalence (liana–tree basal area ratio, woody vine basal area and stem density and rattan stem density). Liana prevalence decreased with elevation but increased with disturbance and mean annual precipitation. The increase in the liana–tree ratio with precipitation was more pronounced for highly disturbed sites. Like other tropical regions, disturbance is an important driver of liana prevalence in Australian rainforests and appears to interact with climate to increase liana–tree ratios. The observed increase in liana–tree ratio with precipitation contrasts findings from elsewhere but is confounded by correlated changes in elevation and temperature, which highlights the importance of regional studies. Our findings show that forests with high disturbance and climatic conditions favourable to lianas are where lianas most likely to outcompete trees and impede forest recovery.

## Graphical abstract

Lianas (woody vines and climbing monocots) are highly important in tropical forests; however, our understanding of the environmental variables that drive their proliferation at regional scales is incomplete and geographically biased. We address this gap through a study in the Australian Wet Tropics and find that disturbance and climate are important drivers of liana prevalence. This provides further support for the concern that global change may be contributing to increasing lianas relative to trees, with serious consequences for rates of tropical forest recovery and carbon sequestration.

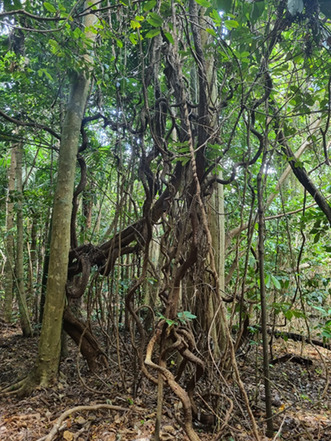

## 1 Introduction

Lianas (woody vines and climbing monocots) play vital roles in forests and are most abundant and diverse in tropical lowland forests (Schnitzer et al. 2002) where they can constitute up to 35% of stem density (Schnitzer et al. 2012), 44% of plant species diversity (Gentry 1991; Pérez‐Salicrup, Sork, and Putz 2001) and 40% of forest leaf area (Hegarty 1991). Lianas root in the ground, use trees for structural support to access the canopy and compete intensely with trees for light, water and nutrients while causing them mechanical stress (Schnitzer, Kuzee, and Bongers 2005; Tobin et al. 2012).

Liana adaptations, such as preference for light (Schnitzer and Bongers 2011), flexible stems (Putz 1984), rapid growth rates (Wyka et al. 2013) and extensive root systems (Putz 2023), often allow them to out‐grow trees in disturbed environments (Buckton et al. 2019). Lianas are known to reduce tree growth (e.g., van der Heijden and Phillips 2009; Ingwell et al. 2010; Estrada‐Villegas et al. 2020), fecundity (García León et al. 2018), survival (McDowell et al. 2018) and recruitment (Martínez‐Izquierdo et al. 2016). Given these effects on trees, liana proliferation can slow forest recovery after disturbances (e.g., Schnitzer, Dalling, and Carson 2000; Schnitzer and Carson 2010; Tymen et al. 2016). Because rates of liana carbon storage are not sufficient to compensate for their effects on trees, lianas can reduce the net carbon uptake of tropical forests, with profound implications for the global carbon sink (van der Heijden, Powers, and Schnitzer 2015; Di Porcia e Brugnera et al. 2019).

Lianas are increasing in abundance relative to trees in many tropical forests (e.g., Phillips et al. 2002; Schnitzer and Bongers 2011; Abiem, Kenfack, and Chapman 2023) with uncertain effects on forest functions. The observed global increases in liana abundance are mostly attributed to increasing anthropogenic disturbance and climate change (Schnitzer et al. 2021; Ngute et al. 2024). At regional scales, however, relationships between liana abundance and environmental variables, such as forest structure, climate, soil and topography, are inconsistent, with studies from Latin America dominating the literature (Schnitzer, Van Der Heijden, and Powers 2016; Estrada‐Villegas et al. 2020).

Natural (e.g., treefalls, cyclones) and anthropogenic disturbances (e.g., selective logging) are important drivers of liana proliferation (e.g., van der Heijden and Phillips 2008; Dalling et al. 2012; Campbell et al. 2017, 2018) but climate also influences lianas. In particular, temperature appears to affect liana distributions with lianas peaking in abundance in the warm, tropical lowlands (Schnitzer 2005). Within the tropics, liana abundance also reportedly decreases with increasing mean annual precipitation and increases with dry season length (Swaine and Grace 2007; Parolari et al. 2020; DeWalt et al. 2010; Ngute et al. 2024), but some studies report no relationship between precipitation and liana abundance in Latin America (van der Heijden and Phillips 2008; Reis et al. 2020) and one study found a positive relationship between lianas and precipitation at a global scale (Durigon, Durán, and Gianoli 2013). Interactions between climate and disturbance may also be important. A recent global meta‐analysis reported that liana dominance increased with time since disturbance in forests with the liana‐favouring conditions of high mean annual temperature and low mean annual precipitation (Ngute et al. 2024), supporting earlier hypotheses (Marshall et al. 2020) and implying that interactions between climate and disturbance need to be better accounted for in future studies.

Topographic variables, especially as they relate to exposure to strong winds, may help explain liana abundance. For example, in regions where strong winds or cyclones occur, wind‐exposed sites are expected to suffer more disturbance (Negrón‐Juárez et al. 2014; Turton and Alamgir 2015), meaning that aspect may show a relationship with liana abundance. Furthermore, lianas decrease in density and richness with increasing elevation (e.g., Jiménez‐Castillo, Wiser, and Lusk 2007; Fadrique and Homeier 2016), most likely because of their association with warmer temperatures. Wide vessels make lianas prone to freeze–thaw embolisms, so they do not grow well at lower temperatures and may be killed by freezing temperatures that can occur at higher elevations (Ewers, Fisher, and Fichtner 1991). Lianas are also reportedly less common on steep slopes (Dalling et al. 2012; Addo‐Fordjour, Rahmad, and Shahrul 2014; but see Nakada et al. 2024).

Previous studies from across the tropics report contrasting results on the relationships between lianas and soils. Some past studies show increases in liana abundance with soil fertility (e.g., Gentry 1991; Putz and Chai 1987; DeWalt et al. 2006; Tymen et al. 2016). Similarly, other studies linked increasing liana abundance, biomass and photosynthetic performance to increased phosphorus, pH, Mg^2+^, K^+^ and Ca^2+^ (Chettri et al. 2010; Malizia, Grau, and Lichstein 2010; Addo‐Fordjour et al. 2015; Pasquini et al. 2015; Fadrique and Homeier 2016; Liu et al. 2020). In contrast, many studies report weak or no relationships between liana abundance and soil properties (e.g., Macía et al. 2007; van der Heijden and Phillips 2008; Lobos‐Catalán and Jiménez‐Castillo 2019; Reis et al. 2020; Waite et al. 2022).

Pan‐tropical and global studies provide valuable insights into the environmental drivers of liana success (e.g., DeWalt et al. 2010; Estrada‐Villegas et al. 2022; Ngute et al. 2024). However, as liana–tree dynamics likely reflect regional conditions, evolutionary histories and disturbance regimes (Corlett and Primack 2006), patterns at regional scales may differ. Given the spatial bias in liana research towards the Neotropics (Schnitzer, van der Heijden, and Powers 2016), studies from other regions are needed that account for these factors (Marshall et al. 2020; Schnitzer et al. 2021). Furthermore, local effects can also be ‘diluted’ at larger scales, therefore global findings may have limited applicability at the regional and local scales that are more relevant to land managers. For example, at global scales, climate is often the main driver of vegetation patterns whereas within regions, topography and edaphic factors may be more important in shaping vegetation dynamics (e.g., van der Heijden and Phillips 2008). There is a notable gap in research on liana–environmental driver relationships from the Australian Wet Tropics (DeWalt et al. 2014). Given that Australian forests show almost zero floristic overlap with the Neotropics (Chave et al. 2019), studies in this region could provide novel insights into the ecology of lianas.

Australian and Asian forests differ from those in the Neotropics in that rattans (climbing palms of the subfamily Calamoideae) are often abundant, whereas they are seldom abundant in the Neotropics. Rattans are typically included in liana inventories (Gerwing et al. 2006). Although they are climbing plants with thick ‘woody’ stems, as monocots, rattans lack the capacity for secondary growth and, therefore, lack true wood (Isnard and Silk 2009). Furthermore, the rattans of the Australian Wet Tropics produce stems that do not branch and emerge from multi‐stemmed clonal clumps (Putz 1990). Hence, rattans can be considered biologically distinct from truly woody lianas. It therefore makes sense to consider rattans separately, while still including them in liana inventories. In this paper, the term woody vine is used to refer to dicot lianas, the term rattan refers to climbing palms of the subfamily Calamoideae, while liana refers to all true woody vines and climbing monocots. Compared to woody vines, relatively little is known about rattans and other climbing monocots and they have been overlooked in previous vegetation studies in Australia, despite their abundance, due in part to challenges in their measurement (Cox et al. 2019). Literature that is available on the response of rattans to environmental variables shows contrasting results (e.g., Siebert 1993; Stiegel et al. 2011; Thonhofer et al. 2015), so there is a need for further study.

Here we test the relative effects of disturbance, climate, topography and soil on lianas in the Australian Wet Tropics. As measures of liana prevalence, we report the abundance of both woody vines and rattans and also report liana–tree ratios (the ratio of liana to tree basal area) (Marshall et al. 2020). Our objectives were to quantify the relative effects of these environmental variables on measures of liana prevalence, to use the findings to identify areas vulnerable to liana proliferation and to discuss the implications of liana proliferation for forest management and global change.

## 2 Materials and Methods

### 2.1 Study Area

We sampled rainforest along an elevational gradient (40–1320 m above sea level) within the Wet Tropics World Heritage Area of northeast Queensland, Australia in an area stretching from the Cassowary Coast Region up to the Atherton Tablelands. The region is a biodiversity hotspot with a globally significant number of endemic species across several taxa (Le Saout et al. 2013). The climate has a pronounced January–April wet season with annual rainfall ranging from 1400 to 3090 mm across the study region (Australian Bureau of Meteorology 2024). The region is prone to tropical cyclones that mostly occur during the wet season and have shaped the structure and composition of these forests (Webb 1958; Mackintosh et al. 2024), most recently by Tropical Cyclone Larry in 2006 (Turton 2008) and Tropical Cyclone Yasi in 2011 (Negrón‐Juárez et al. 2014). The forests are also heavily fragmented due to land clearing for agriculture and urban development since European settlement in the early 1900s (Winter, Bell, and Pahl 1987). Most of the remaining forest was also selectively logged until the region received World Heritage status in 1988 (Goosem and Tucker 2013). Specific details on the location and extent of logging are not available following the closure of the forestry industries in the region.

### 2.2 Data Collection

**FIGURE 1:**
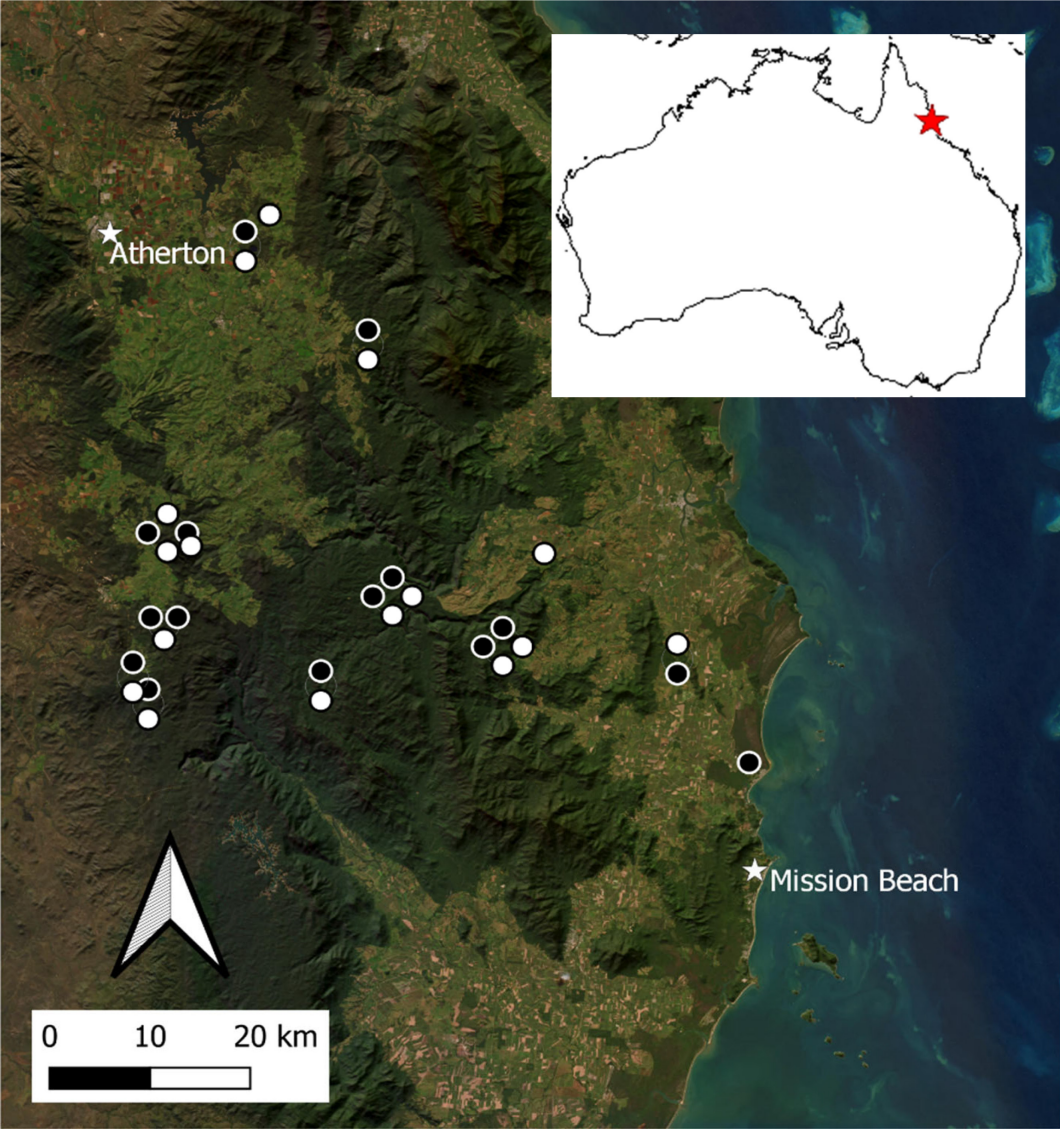
Location of 31 vegetation plots (20 × 20 m) stratified at approximately 100 m elevation intervals across an elevation gradient (40–1320 m) in the Wet Tropics region of northeast Queensland, Australia. Black circles indicate less disturbed plots (*n* = 15) and white circles indicate highly disturbed plots (*n* = 16). (Nearby plots are displaced slightly for visibility). Map created using QGIS v3.22.

Thirty‐one 20 × 20 m vegetation plots were established at approximately 100 m elevational intervals along the 40–1320 m elevation gradient in pairs, with one plot ‘highly disturbed’ and the other ‘less disturbed’ (except for around 800 m elevation where, due to time constraints, there is only a ‘highly disturbed’ plot; Figure 1). Less disturbed plots were defined as having ≥ 75% cover by trees ≥ 5 m tall and no signs of logging. Highly disturbed plots were defined as having only ≤ 25% cover by trees ≥ 5 m tall. There is no old‐growth forest in the region due to widespread past cyclone disturbance. Given the absence of detailed logging records and the region's past exposure to multiple cyclones with varying intensities, wind paths and intervals, it is not possible to verify the exact disturbance history of each plot, including the type of disturbance and the time since it occurred.

In each plot, the stem diameters of all trees and lianas ≥ 1 cm DBH (diameter at breast height; 1.30 m) were measured and tagged following standard protocols (Gerwing et al. 2006; Schnitzer, Rutishauser, and Aguilar 2008; Marthews et al. 2014). Lianas were noted as either woody vines, rattans or other families of climbing monocot. Elevation was measured using a Garmin GPSMAP 64sx. Slope and aspect data were collected using a clinometer and compass. Soil samples (0–30 cm) were collected from the centre of each plot using a hand auger, after removing any leaf litter. All field data were collected between August 2021 and November 2022.

At the plot level, we calculated the liana–tree ratio as the basal area of lianas relative to the basal area of trees. Basal area (cm^2^) and stem density (number of stems per 20 × 20 m plot) were also calculated separately for woody vines and rattans. Rattan basal area and stem density were highly correlated (*r* = 0.9) so only rattan stem density is included in the analysis. Other climbing monocots (Flagellariaceae, Smiliaceae and Poaceae) were also recorded and included in the liana–tree ratio but were too few for separate analyses. We used basal area instead of biomass because calculations of the biomass of rattans and other monocots require data for both stem diameter and length and we lack the latter. We also tested the relationship between (1) woody vine basal area and rattan basal area and (2) woody vine stem density and rattan stem density, with the expectation that these measures would be positively correlated as both plant forms occupy similar ecological niches.

Soil samples were air‐dried and sieved through a 2 mm mesh. Soil pH was determined using a pH meter in a 1:5 soil‐to‐water ratio using a LABCHEM pH meter. To measure total phosphorous (P), samples of soil were digested with a mixture of 70% nitric acid and 70% perchloric acid in a temperature‐controlled digestion block and then the digested samples were analysed by Inductively Coupled Plasma‐Optical Emission Spectrometry. To measure exchangeable bases (Na, Mg, Ca and K), samples were first extracted with 1 M ammonium chloride at pH 7.0 and then analysed with Inductively Coupled Plasma Mass Spectrometry (Agilent 8900 ICP‐MS). These values were then summed to obtain a measure of total exchangeable bases.

To obtain climate data, we used bioclimatic variables from the WorldClim V2.1 database at 30‐arc‐second spatial resolution (~1 × 1 km). WorldClim data are derived using observations from local weather stations, which are then interpolated using additional covariates to create a climate surface (Fick and Hijmans 2017). For each plot location, we extracted variables from the WorldClim climate surface that were identified as important drivers of liana abundance in previous studies (DeWalt et al. 2010; Ngute et al. 2024): minimum temperature, mean annual precipitation and precipitation seasonality.

### 2.3 Statistical Analysis

To assess the environmental drivers of liana prevalence, we tested the relationship between environmental variables (climate, soil, topography and disturbance) and measures of liana prevalence: liana–tree ratio, woody vine basal area, woody vine stem density and rattan stem density. Prior to modelling, the aspect was cosine transformed to remove circularity and give a measure of ‘northness’ and sine transformed to give a second measure of ‘eastness’ (Roberts and Cooper 1989). The predictor variables were tested for multicollinearity using pairwise Pearson correlation coefficients and variance inflation factors (VIF). For high correlations (*r* ≥ 0.7) and VIF ≥ 4, the variable with the weakest correlation with the response variable was dropped from subsequent multivariate modelling.

The remaining predictor variables were used in generalised linear models (GLMs) to compare each measure of liana prevalence against multiple predictors. An interaction term between climate variables and disturbance was introduced to test the hypothesis that disturbance has a stronger effect on lianas under liana‐favouring climatic conditions (Marshall et al. 2020). Predictor variables were standardised to allow comparison of effect sizes (Grueber et al. 2011) using the ‘scale’ function in R. Liana–tree ratio and woody vine basal area were both modelled using a gamma error distribution and log link because they were non‐negative and positively skewed. Stem densities of woody vines and rattans were modelled using a negative binomial GLM because of significant overdispersion in an initial Poisson model used for count data (Zuur et al. 2010). For each response variable, we first constructed a full model containing all the uncorrelated predictors and potential interaction effects. For each full model, we used the ‘dredge’ function of the ‘MuMIn’ package (Barton 2019) to list all possible model combinations and their Corrected Akaike Information Criterion (AIC_c_) score. The ‘model.avg’ function was used to obtain a final model, which was the averaged model calculated from a subset of models where ΔAIC_c_ < 2 (Burnham and Anderson 2002).

We also calculated the relative importance of each variable retained in the final model by summing model weights for all models in the subset ΔAIC_c_ < 2 where that specific variable was included. Relative variable importance has a maximum of 1, which would mean that the variable appeared in every model in the subset.

Final model fits, based on the unstandardised data, were predicted using the ‘predict’ function, with 95% confidence intervals calculated around the fitted model. In this case, the raw, unstandardised data were used to ease interpretation. For each final model, plots were created for each retained predictor, visualising the slope of the prediction where that predictor was allowed to vary while the others were held constant at their mean. This approach allows visualisation of the individual effect of each variable in the final model by removing the potential influence of the other variables.

The residuals of each final model were calculated and checked for spatial autocorrelation using Moran's *I* (Moran 1950) in the ‘spdep’ package. For this analysis, 5 km was chosen as the distance to include neighbours as this was roughly the largest fragment size for plots located in fragmented forests. All statistical analyses were conducted using R v4.2.0 (R Core Team 2022).

## 3 Results

**FIGURE 2:**
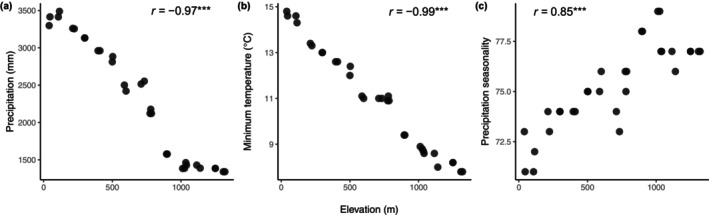
Strong intercorrelation between elevation and climate predictors: (a) mean annual precipitation (Pearson correlation coefficient = −0.97); (b) minimum temperature (Pearson correlation coefficient = −0.99) and (c) precipitation seasonality (Pearson correlation coefficient = 0.85). Asterisks indicate significance levels (*p* < 0.001***).

**FIGURE 3:**
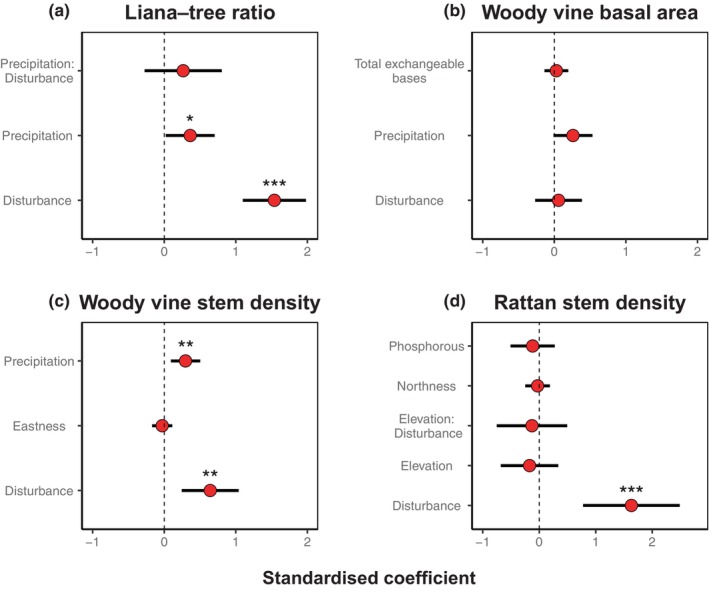
Coefficient plots for environmental predictors in each final model explaining: (a) liana–tree ratio; (b) woody vine basal area; (c) woody vine stem density; (d) rattan stem density (calculated from the subset of models where ΔAIC_c_ < 2). Points indicate standardised coefficients with 95% confidence intervals. Asterisks indicate level of significance (*p < 0.05, **p < 0.01, ***p < 0.001).

**FIGURE 4:**
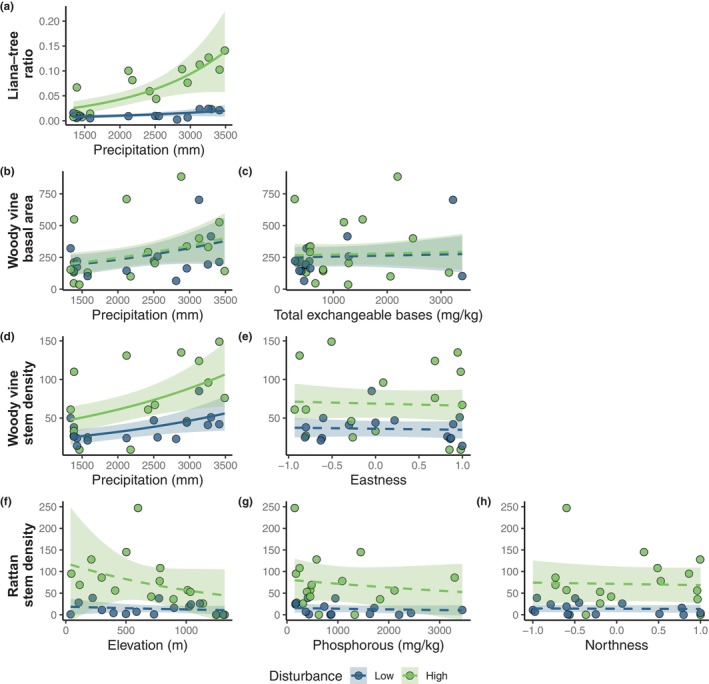
Modelled relationships between measures of liana prevalence: (a) liana–tree ratio, (b) and (c) woody vine basal area (cm^2^); (d) and (e) woody vine stem density (per 20 × 20 m plot) and (f–h) rattan stem density (per 20 × 20 m plot) and environmental predictors. Each plot shows the fit of each predictor retained in each final model (calculated as the average of a subset of models where ΔAIC_c_ < 2), while other predictors are held constant (set to their means) with 95% confidence intervals. Solid lines indicate significant relationships (*p* < 0.05).

**FIGURE A1:**
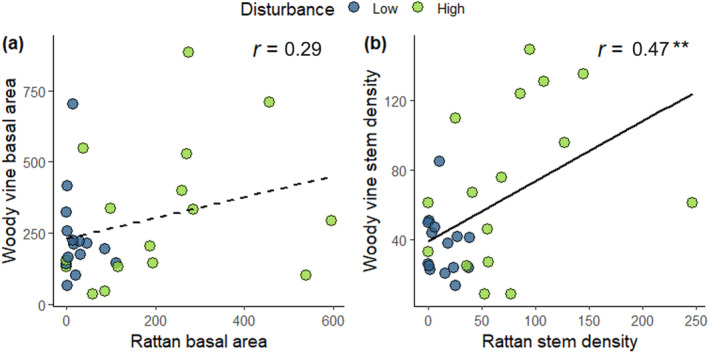
Relationship between (a) woody vine basal area and rattan basal area (Pearson correlation coefficient = 0.29) and (b) woody vine stem density and rattan stem density (Pearson correlation coefficient = 0.47). Lines of best fit are presented on graphs. Solid line indicates a significant relationship (*p* < 0.05). Asterisk indicates the significance level (*p* < 0.01**).

Elevation, precipitation, minimum temperature and precipitation seasonality were all highly intercorrelated (*r* ≥ 0.85) (Figure 2). Only precipitation was retained in the final models for the liana–tree ratio, woody vine basal area and woody vine stem density while elevation was retained in the final model for rattan stem density. Variable importance for the predictors retained in each of the final models is presented in Table 1, standardised coefficient plots in Figure 3 and plots visualising the effects of each individual predictor in the final models in Figure 4. Further details on the subset of models used to calculate the final averaged models are presented in Table A1 in Appendix A. Rattan basal area and woody vine basal area were not correlated, but rattan stem density and woody vine stem density were positively correlated (Figure A1 in Appendix A).

All measures of liana prevalence increased with disturbance with a maximum variable importance of 1 for liana–tree ratios, woody vine stem densities and rattan stem densities. In contrast, in the final model for the woody vine basal area, the disturbance had an importance of only 0.24 and was not significant (Table 1; Figure 3).

**TABLE 1:** Summary of variable importance for each variable retained in final models quantifying the relationships between measures of liana prevalence (liana–tree ratio, woody vine basal area, woody vine stem density, rattan stem density) and standardised predictor variables. The final models are based on an averaged model calculated from a subset of models with ΔAIC_c_ < 2.

Response	Predictors	Variable importance
Liana–tree ratio	Precipitation	1.00
	Disturbance	1.00
	Precipitation: Disturbance	0.63
Woody vine basal area	Precipitation	1.00
	Disturbance	0.24
	Total exchangeable bases	0.23
Woody vine stem density	Precipitation	1.00
	Disturbance	1.00
	Eastness	0.28
Rattan stem density	Disturbance	1.00
	Elevation	0.53
	Phosphorous	0.38
	Northness	0.19
	Elevation: Disturbance	0.11

Liana–tree ratio increased with precipitation and this variable ranked as most important in the final model, alongside disturbance. Additionally, there was an interaction between disturbance and precipitation (Figure 3), which was the third most important variable in explaining liana–tree ratio (Table 1), appearing in the top‐ranked model (Table A1 in Appendix A). The positive effect of precipitation on liana–tree ratios was much stronger in plots with high disturbance (Figure 4), but this interaction term was not statistically significant . Precipitation was also the most important predictor in the final models explaining woody vine basal area and woody vine stem density (Table 1). Total exchangeable bases appeared in the final model explaining the woody vine basal area, but this was not significant and had minimal effect compared to climate (Figures 3 and 4).

Apart from disturbance, all other predictors retained the final rattan model were not statistically significant. After the disturbance, elevation was ranked as the most important variable in the final model for rattan stem density, with a decrease in rattan stems with increasing elevation. This trend was driven by the relationship across plots with high disturbance and accordingly, the interaction effect between elevation and disturbance was retained in the final model (Table 1; Figures 3 and 4). Soil phosphorous and northness also appeared in the final model for rattan stem density but were not as influential as elevation and disturbance (Table 1; Figure 3).

The topographic variables of slope, northness and eastness had little effect on all measures of liana prevalence, with relatively low importance in all the final models, or not appearing at all, suggesting slope and aspect did not strongly influence any measures of liana prevalence (Table 1). The Moran's *I* test indicated that no spatial autocorrelations were present in the final models (Table 2).

**TABLE 2:** Results of Moran's *I* test on residuals of each final model indicating the lack of spatial autocorrelations.

	Moran's *I*	*p*
Liana–tree ratio	0.139	0.154
Woody vine basal area	−0.236	0.879
Woody vine stem density	0.181	0.794
Rattan stem density	0.038	0.310

## 4 Discussion

We tested the relative effects of disturbance, climate, topography and soil on lianas in the Australian Wet Tropics, a region that has been underrepresented in the liana literature. We found disturbance had the strongest influence on most measures of liana prevalence, followed by climate. The interaction between disturbance and climate was also important for the liana–tree ratio. Soil and aspect had negligible effects on liana prevalence compared to disturbance and climate. These findings can be used to identify areas that may be most vulnerable to liana proliferation and thus should be a priority for management.

Our findings lend further support to the suggestion that disturbance is the primary driver of increasing liana dominance in tropical forests (Marshall et al. 2020; Schnitzer et al. 2021). While disturbance increased liana–tree ratios as well as densities of woody vines and rattans, it had relatively less effect on woody vine basal area. This finding, which agrees with those previously reported (van der Heijden and Phillips 2008), may be explained by lianas in old‐growth forests growing larger while declining in density (Poulsen et al. 2017; Campbell et al. 2018). Moreover, lianas grow slowly in diameter (Putz 1990; Isnard and Silk 2009) and therefore lacked time to grow large in recently disturbed plots. The absence of a relationship between woody vine basal area and disturbance suggests the relationship between disturbance and the liana–tree ratio is driven by rattans and/or trees. Both are likely important; the highly disturbed plots contain fewer large trees compared to lightly disturbed plots and our results show that rattans were more abundant in these highly disturbed plots.

In addition to disturbance, liana–tree ratios and woody vine basal areas and stem densities increased with precipitation, which contradicts previous reports of the opposite trend (e.g., Schnitzer 2005; DeWalt et al. 2010; Ngute et al. 2024). It may be important to note that these other studies were conducted at continental and global scales and encompassed a much wider range of precipitation (~0–7500 mm) whereas across our plots, precipitation ranged only 1340–3489 mm. In our study landscape, rainfall decreases from southwest to northeast due to increasing elevation from the coast up onto the Atherton Tablelands (Unwin and Kriedemann 1990). Elevation therefore also correlates with decreasing minimum temperatures and increasing precipitation seasonality, which explains the multicollinearity between elevation and these climatic variables (Figure 2).

It is difficult to ascertain which of the climate variables is most important given the high correlation between them. Overall, it is likely that the observed increase in liana–tree ratio with precipitation is driven by elevation and/or minimum temperature, rather than by precipitation directly. It is well established that lianas decrease in abundance (Bruy et al. 2017), biomass (Alves et al. 2012; Fadrique and Homeier 2016), species richness (Webb 1968) and liana–tree ratios (Ngute et al. 2024) with increasing elevation, presumably as a result of decreasing temperatures (Schnitzer et al. 2002). In addition to climate, local disturbance regimes may help explain the increased liana prevalence at lower elevations, where rainfall is highest. Due to their coastal proximity, the lowlands suffered more extensive cyclone damage (Negrón‐Juárez et al. 2014; Turton 2019), which promoted liana proliferation and tree mortality (Metcalfe, Bradford, and Ford 2008; Murphy and Metcalfe 2016). Our finding of increasing precipitation with measures of liana prevalence highlights the limitations of applying results from global studies to regional or local scales, which are often the scale most relevant to land managers. Global studies ‘dilute’ local effects and, therefore, often fail to recognise them.

Although WorldClim data are generally regarded as reliable (Fick and Hijmans 2017), there are important limitations to acknowledge. The bioclimatic variables are derived from interpolated data from local weather stations, which are unevenly distributed across the region, introducing a degree of uncertainty. Additionally, the 1 × 1 km grid lacks fine spatial resolution, which can result in inaccuracies when predicting climate across heterogeneous terrain. For instance, in mountainous regions like our study area, elevation and topography—and consequently temperature and rainfall—can vary substantially within < 1 km (Poggio, Simonetti, and Gimona 2018). In future studies, plot‐level climate data may provide more insights.

The observed interaction between precipitation and disturbance indicates that in highly disturbed sites where lianas are abundant, climate may have a strong effect. This finding is consistent with a recent global meta‐analysis that found disturbance had a stronger influence on liana–tree ratios under climatic conditions favourable to lianas (Ngute et al. 2024). While important in the final model, it should be noted that this interaction term was not significant, so further study would be needed to increase confidence in this finding. Nevertheless, this finding suggests that disturbance may interact with climatic variables to increase liana–tree ratios. There is concern that increasing disturbances and climate change could interact to intensify liana prevalence, with negative consequences for forests, making further study of utmost importance (Marshall et al. 2020).

Disturbance aside, none of the predictors in the final model for rattans appeared to be strong predictors of their density. Rattan density decreased with elevation, but this relationship was not significant, contrasting with past studies from Indonesia that reported that rattan density peaks at 1000–1100 m (Watanabe and Suzuki 2008; Stiegel et al. 2011). This finding highlights that Australian rattan species may not exhibit the same trends as those native to Southeast Asia. Rattans are widely harvested as a non‐timber forest product in Southeast Asia (Ros‐Tonen 2000) but not in our study site. Harvesting pressure at lower elevations in Asia may, therefore, explain the differences observed between the two regions (Stiegel et al. 2011). In addition, we found a slight decrease in rattan stem density with soil phosphorous, but its effect was negligible compared to elevation and disturbance. Previous findings from Southeast Asia showed that rattan richness and density were influenced by soil pH, K and Ca, but not phosphorous (Thonhofer et al. 2015; Rozali et al. 2021).

Of the measured soil parameters, total exchangeable bases was retained in the final model for the woody vine basal area, but it had negligible effect compared to precipitation. This finding agrees with many previous studies that found no influence of soil on lianas at both landscape and broader geographical scales (e.g., Balfour and Bond 1993; DeWalt and Chave 2004; van der Heijden and Phillips 2008; Reis et al. 2020; Waite et al. 2022) as well as in nutrient addition experiments (Pasquini et al. 2015; Schnitzer, Estrada‐Villegas, and Wright 2020). Due to their nutrient‐rich foliage (Asner and Martin 2012) and extensive root systems (Collins, Wright, and Wurzburger 2016; Smith‐Martin et al. 2019; Putz 2023), lianas have a reciprocal relationship with soil, responding to soil properties but also influencing them, which can be difficult to disentangle (Powers 2015). Furthermore, soil nutrient levels directly and indirectly affect many other forest structural attributes and processes (e.g., Phillips et al. 2004; Cusack et al. 2016) that may obscure any impacts on lianas. Overall, interactions between lianas and soil are likely complex and warrant further study.

Slope did not have a strong influence on the prevalence of lianas in any of the final models. This finding contrasts with some previous studies that found lianas were less common on slopes of similar steepness to those reported in our study (Dalling et al. 2012; Addo‐Fordjour, Rahmad, and Shahrul 2014) and another that reported lianas were more common on steep slopes (Nakada et al. 2024). Our findings agree with past studies showing that slope had minimal influence on liana abundance (Kusumoto, Enoki, and Watanabe 2008; Lertpanich and Brockelman 2003; Waite et al. 2022; Ngute et al. 2024). We expected that aspect would be related to liana prevalence due to varying exposure to cyclonic winds and found that while eastness and northness appeared in final models for woody vine and rattan stem densities, respectively, their effects were minor compared to climate and disturbance. Cyclone paths can be difficult to map and vary with topography in non‐uniform ways (Ramsay and Leslie 2008; Turton 2008); therefore across the wider landscape, it may not always be a consistent aspect that is most strongly affected by cyclone damage, which would dilute any potential effects at this level.

Liana cutting can be an effective management tool for promoting tree growth and carbon sequestration (Finlayson et al. 2022; Putz et al. 2023) and further research on this topic is considered essential for the effective restoration of forests worldwide (Marshall et al. 2023). The benefits of liana cutting notwithstanding, they do contribute to biodiversity (e.g., Odell, Stork, and Kitching 2019; Schnitzer, Estrada‐Villegas, and Wright 2020; Schnitzer et al. 2020) and aspects of rainforest functioning, such as nutrient cycling (Tang, Kitching, and Cao 2012; Campbell et al. 2015) and may even provide protective ‘bandage effects’ (Marshall et al. 2020). Hence, widespread liana removal could have unanticipated negative effects. With knowledge of the environmental conditions that drive liana dominance, practitioners can focus on cutting lianas where they are likely to have the greatest detrimental effects. Directing management to specific areas could minimise any potential adverse effects that may result from widespread liana removal. Our findings highlight that in our study region of the Australian Wet Tropics, the heavily disturbed areas of the wetter, warmer lowlands might be a priority area for this management intervention. However, given our limited sample size and single timeframe, further study is advised before widespread liana cutting should be implemented. These studies should track whether lianas persist in heavily disturbed areas, in which case, cutting at least some of them may be a necessary intervention to promote biomass recovery.

Understanding the environmental drivers of liana prevalence can inform predictions about the future of tropical forests and carbon storage under different climate change scenarios, especially given that climate change is likely contributing to increases in liana abundance in the study region (Vogado et al. 2022). Based on our findings, climate change and increasing anthropogenic disturbances may interact to increase liana dominance, with serious consequences for the recovery of tropical forests and their ability to sequester carbon. Given tropical forest regrowth is expected to play a role in mitigating climate change (e.g., Chazdon et al. 2016; Heinrich et al. 2021), this is concerning and further highlights the importance of further research on these heavily disturbed and liana‐dominated sites.

## 5 Conclusion

Our findings enhance the understanding of liana ecology and provide an Australian study to counter the current Neotropical bias. Overall, we found that lianas were most dominant in heavily disturbed sites in the warmer, wetter lowlands and that climate and disturbance interact to increase liana–tree ratios. At a regional scale, these findings have management implications, informing practitioners of where liana cutting may have benefits for trees. At a global scale, in light of increasing temperatures due to climate change and ongoing disturbances to forests, these findings indicate that lianas may continue to increase relative to trees with uncertain consequences for forest functioning, including carbon sequestration.

## Author Contributions

**Emma J. Mackintosh:** conceptualization (equal), data curation (lead), formal analysis (lead), investigation (lead), methodology (equal), project administration (equal), resources (equal), visualization (lead), writing – original draft (lead). **Andrew R. Marshall:** conceptualization (equal), formal analysis (supporting), funding acquisition (lead), methodology (equal), project administration (equal), resources (equal), supervision (equal), validation (equal), writing – review and editing (equal). **Sophie Brennan:** data curation (supporting), investigation (equal), resources (supporting), writing – review and editing (supporting). **Zhongming Lan:** methodology (supporting), resources (equal), validation (equal), writing – review and editing (supporting). **Marion Pfeifer:** conceptualization (equal), formal analysis (supporting), funding acquisition (supporting), methodology (supporting), supervision (equal), validation (equal), writing – review and editing (equal). **Catherine E. Waite:** formal analysis (supporting), methodology (supporting), supervision (equal), validation (equal), writing – review and editing (equal). **Chengrong Chen:** methodology (supporting), resources (equal), validation (equal), writing – review and editing (supporting). **Francis E. Putz:** supervision (equal), validation (equal), writing – review and editing (equal).

## Conflicts of Interest

The authors declare no conflicts of interest.

## Data Availability

All data used in this study is openly available at: https://datadryad.org/stash/share/2BZodMvI8tCNggnVJcxJBQ9S‐PHA6xjA243T9r4rT‐Q.

## Appendix A

**TABLE A1:** List of the subset models with ΔAIC_c_ < 2 used to calculate the final averaged model for each measure of liana prevalence: Liana–tree ratio, woody vine basal area, woody vine stem density and rattan stem density.

Response	Model	Disturbance	Precipitation	Precipitation: Disturbance	Total exchangeable bases	Eastness	Northness	Phosphorous	Elevation	Elevation: Disturbance	Pseudo‐*R* ^2^	AIC_c_	ΔAIC_c_	Weight
Liana–tree ratio	1	1.54***	0.29	0.42							0.84	−169.3	0	0.63
	2	1.54***	0.48***								0.81	−168.23	1.08	0.37
Woody vine basal area	1		0.27								0.16	404.9	0	0.53
	2	0.25	0.26*								0.20	406.45	1.55	0.24
	3		0.24		0.13						0.19	406.53	1.63	0.23
Woody vine stem density	1	0.64**	0.29**								0.59	296.35	0	0.72
	2	0.65***	0.31**			−0.1					0.62	298.21	1.86	0.28
Rattan stem density	1	1.67***									0.46	287.91	0	0.23
	2	1.56***							−0.37		0.52	288.38	0.47	0.18
	3	1.54***						−0.33	−0.48*		0.60	288.67	0.76	0.16
	4	1.70***						−0.23			0.50	289.16	1.24	0.12
	5	1.88***					−0.26				0.49	289.36	1.45	0.11
	6	1.50***						−0.36	−0.18	−0.71	0.65	289.63	1.72	0.1
	7	1.55***							−0.11	−0.62	0.60	289.68	1.77	0.1

*Note:* Standardised coefficient estimates are quoted for each variable in the model. Asterisks indicate significance levels (*p* < 0.05*, *p* < 0.01**, *p* < 0.001***). Pseudo‐*R* ^2^ (Nagelkerke 1991), AIC_c_, ΔAIC_c_ and AIC_c_ weights are also reported. Nagelkerke's pseudo‐*R* ^2^ indicates the proportion of variance explained by the model.

## References

[ece370374-bib-0001] Abiem, I. , D. Kenfack , and H. M. Chapman . 2023. “Assessing the Impact of Abiotic and Biotic Factors on Seedling Survival in an African Montane Forest.” Frontiers in Forests and Global Change 6: 1108257. 10.3389/ffgc.2023.1108257.

[ece370374-bib-0002] Addo‐Fordjour, P. , Z. B. Rahmad , and A. M. S. Shahrul . 2014. “Environmental Factors Influencing Liana Community Diversity, Structure and Habitat Associations in a Tropical Hill Forest, Malaysia.” Plant Ecology and Diversity 7, no. 4: 485–496. 10.1080/17550874.2013.782369.

[ece370374-bib-0113] Addo‐Fordjour, P. , and Z. B. Rahmad . 2015. “Environmental Factors Associated With Liana Community Assemblages in a Tropical Forest Reserve, Ghana.” Journal of Tropical Ecology 31, no. 1: 69–79. 10.1017/S0266467414000522.

[ece370374-bib-0121] Alves, L. F. , M. A. Assis , J. van Melis , et al. 2012. “Variation in Liana Abundance and Biomass Along an Elevational Gradient in the Tropical Atlantic Forest (Brazil).” Ecological Research 27, no. 2: 323–332. 10.1007/s11284-011-0902-8.

[ece370374-bib-0124] Asner, G. P. , and R. E. Martin . 2012. “Contrasting Leaf Chemical Traits in Tropical Lianas and Trees: Implications for Future Forest Composition.” Ecology Letters 15: 1001. 10.1111/j.1461-0248.2012.01821.x. 22690783

[ece370374-bib-0117] Australian Bureau of Meteorology . 2024. “Climate Data Online.” Accessed April 8, 2024. http://www.bom.gov.au/climate/data.

[ece370374-bib-0003] Balfour, A. D. A. , and W. J. Bond . 1993. “Factors Limiting Climber Distribution and Abundance in a Southern African Forest.” Journal of Ecology 81, no. 1: 93–100.

[ece370374-bib-0004] Barton, K. 2019. “MuMIn: Multi‐model inference.” R package version 1.43.6. https://CRAN.R‐project.org/package=MuMIn.

[ece370374-bib-0005] Buckton, G. , A. W. Cheesman , N. C. Munksgaard , C. M. Wurster , M. J. Liddell , and L. A. Cernusak . 2019. “Functional Traits of Lianas in an Australian Lowland Rainforest Align With Post‐Disturbance Rather Than Dry Season Advantage.” Austral Ecology 44, no. 6: 983–994. 10.1111/aec.12764.

[ece370374-bib-0120] Bruy, D. , T. Ibanez , J. Munzinger , and S. Isnard . 2017. “Abundance, Richness and Composition of Lianas in Forest Communities Along an Elevation Gradient in New Caledonia.” Plant Ecology and Diversity 10, no. 5–6: 469–481. 10.1080/17550874.2018.1430186.

[ece370374-bib-0006] Burnham, K. P. , and D. R. Anderson . 2002. Model Selection and Multimodel Inference: A Practical Information‐Theoretic Approach. New York, NY: Springer‐Verlag.

[ece370374-bib-0007] Campbell, M. J. , W. Edwards , A. Magrach , et al. 2018. “Edge Disturbance Drives Liana Abundance Increase and Alteration of Liana–Host Tree Interactions in Tropical Forest Fragments.” Ecology and Evolution 8, no. 8: 4237–4251. 10.1002/ece3.3959. 29721294 PMC5916267

[ece370374-bib-0008] Campbell, M. J. , W. Edwards , A. Magrach , et al. 2017. “Forest Edge Disturbance Increases Rattan Abundance in Tropical Rain Forest Fragments.” Scientific Reports 7, no. 1: 6071. 10.1038/s41598-017-06590-5. 28729670 PMC5519600

[ece370374-bib-0009] Campbell, M. J. , W. Edwards , E. Odell , D. Mohandass , and W. F. Laurance . 2015. “Can Lianas Assist in Rainforest Restoration?” Tropical Conservation Science 8, no. 1: 257–273. 10.1177/194008291500800119.

[ece370374-bib-0010] Chave, J. , S. J. Davies , O. L. Phillips , et al. 2019. “Ground Data Are Essential for Biomass Remote Sensing Missions.” Surveys in Geophysics 40, no. 4: 863–880.

[ece370374-bib-0125] Chazdon, R. L. , E. N. Broadbent , D. M. A. Rozendaal , et al. 2016. “Carbon Sequestration Potential of Second‐Growth Forest Regeneration in the Latin American Tropics.” Science Advances 2, no. 5: e1501639. 10.1126/sciadv.1501639. 27386528 PMC4928921

[ece370374-bib-0011] Chettri, A. , S. K. Barik , H. N. Pandey , and M. K. Lyngdoh . 2010. “Liana Diversity and Abundance as Related to Microenvironment in Three Forest Types Located in Different Elevational Ranges of the Eastern Himalayas.” Plant Ecology and Diversity 3, no. 2: 175–185. 10.1080/17550874.2010.495140.

[ece370374-bib-0012] Collins, C. G. , S. J. Wright , and N. Wurzburger . 2016. “Root and Leaf Traits Reflect Distinct Resource Acquisition Strategies in Tropical Lianas and Trees.” Oecologia 180, no. 4: 1037–1047. 10.1007/s00442-015-3410-7. 26254258

[ece370374-bib-0013] Corlett, R. T. , and R. B. Primack . 2006. “Tropical Rainforests and the Need for Cross‐Continental Comparisons.” Trends in Ecology & Evolution 21, no. 2: 104–110. 10.1016/j.tree.2005.12.002. 16701482

[ece370374-bib-0014] Cox, C. J. , W. Edwards , M. J. Campbell , W. F. Laurance , and S. G. W. Laurance . 2019. “Liana Cover in the Canopies of Rainforest Trees is Not Predicted by Local Ground‐Based Measures.” Austral Ecology 44, no. 5: 759–767. 10.1111/aec.12746.

[ece370374-bib-0015] Cusack, D. F. , J. Karpman , D. Ashdown , et al. 2016. “Global Change Effects on Humid Tropical Forests: Evidence for Biogeochemical and Biodiversity Shifts at an Ecosystem Scale.” Reviews of Geophysics 54, no. 3: 523–610. 10.1002/2015RG000510.

[ece370374-bib-0016] Dalling, J. W. , S. A. Schnitzer , C. Baldeck , et al. 2012. “Resource‐Based Habitat Associations in a Neotropical Liana Community.” Journal of Ecology 100, no. 5: 1174–1182. 10.1111/j.1365-2745.2012.01989.x.

[ece370374-bib-0017] DeWalt, S. J. , and J. Chave . 2004. “Structure and Biomass of Four Lowland Neotropical Forests.” Biotropica 36, no. 1: 7–19. 10.1111/j.1744-7429.2004.tb00291.x.

[ece370374-bib-0018] DeWalt, S. J. , K. Ickes , R. Nilus , K. E. Harms , and D. F. R. P. Burslem . 2006. “Liana Habitat Associations and Community Structure in a Bornean Lowland Tropical Forest.” Plant Ecology 186, no. 2: 203–216. 10.1007/s11258-006-9123-6.

[ece370374-bib-0019] DeWalt, S. J. , S. A. Schnitzer , L. F. Alves , et al. 2014. “Biogeographical Patterns of Liana Abundance and Diversity.” In Ecology of Lianas, edited by S. A. Schnitzer , F. Bongers , R. J. Burnham , and F. E. Putz , 131–145. Oxford, UK: Wiley‐Blackwell. 10.1002/9781118392409.ch11.

[ece370374-bib-0020] DeWalt, S. J. , S. A. Schnitzer , J. Chave , et al. 2010. “Annual Rainfall and Seasonality Predict Pan‐Tropical Patterns of Liana Density and Basal Area.” Biotropica 42, no. 3: 309–317. 10.1111/j.1744-7429.2009.00589.x.

[ece370374-bib-0021] Di Porcia e Brugnera, M. , F. Meunier , M. Longo , et al. 2019. “Modelling the Impact of Liana Infestation on the Demography and Carbon Cycle of Tropical Forests.” Global Change Biology 25, no. 11: 3767–3780. 10.1111/gcb.14769. 31310429 PMC6856694

[ece370374-bib-0022] Durigon, J. , S. M. Durán , and E. Gianoli . 2013. “Global Distribution of Root Climbers Is Positively Associated With Precipitation and Negatively Associated With Seasonality.” Journal of Tropical Ecology 29, no. 4: 357–360. 10.1017/S0266467413000308.

[ece370374-bib-0023] Estrada‐Villegas, S. , J. S. Hall , M. van Breugel , and S. A. Schnitzer . 2020. “Lianas Reduce Biomass Accumulation in Early Successional Tropical Forests.” Ecology 101, no. 5: e02989. 10.1002/ecy.2989. 31961451

[ece370374-bib-0024] Estrada‐Villegas, S. , S. S. Pedraza Narvaez , A. Sanchez , and S. A. Schnitzer . 2022. “Lianas Significantly Reduce Tree Performance and Biomass Accumulation Across Tropical Forests: A Global Meta‐Analysis.” Frontiers in Forests and Global Change 4: 232. 10.3389/FFGC.2021.812066/BIBTEX.

[ece370374-bib-0025] Ewers, F. W. , J. B. Fisher , and K. Fichtner . 1991. “Water Flux and Xylem Structure in Vines.” In Biology of Vines, edited by F. E. Putz and H. A. Mooney , 127–160. Cambridge, UK: Cambridge University Press.

[ece370374-bib-0026] Fadrique, B. , and J. Homeier . 2016. “Elevation and Topography Influence Community Structure, Biomass and Host Tree Interactions of Lianas in Tropical Montane Forests of Southern Ecuador.” Journal of Vegetation Science 27, no. 5: 958–968. 10.1111/jvs.12427.

[ece370374-bib-0027] Fick, S. E. , and R. J. Hijmans . 2017. “WorldClim 2: New 1‐Km Spatial Resolution Climate Surfaces for Global Land Areas.” International Journal of Climatology 37, no. 12: 4302–4315. 10.1002/joc.5086.

[ece370374-bib-0028] Finlayson, C. , A. Roopsind , B. W. Griscom , D. P. Edwards , and R. P. Freckleton . 2022. “Removing Climbers More Than Doubles Tree Growth and Biomass in Degraded Tropical Forests.” Ecology and Evolution 12, no. 3: e8758. 10.1002/ece3.8758. 35356565 PMC8948070

[ece370374-bib-0029] García León, M. M. , L. Martínez Izquierdo , F. N. A. Mello , J. S. Powers , and S. A. Schnitzer . 2018. “Lianas Reduce Community‐Level Canopy Tree Reproduction in a Panamanian Forest.” Journal of Ecology 106, no. 2: 737–745. 10.1111/1365-2745.12807.

[ece370374-bib-0030] Gentry, A. H. 1991. “The Distribution and Evolution of Climbing Plants.” In Biology of Vines, edited by F. E. Putz and H. A. Mooney , 3–49. Cambridge, UK: Cambridge University Press.

[ece370374-bib-0031] Gerwing, J. J. , S. A. Schnitzer , R. J. Burnham , et al. 2006. “A Standard Protocol for Liana Censuses.” Biotropica 38, no. 2: 256–261. http://www.jstor.org/stable/30044913.

[ece370374-bib-0032] Goosem, S. , and N. I. J. Tucker . 2013. Repairing the Rainforest. 2nd ed. Cairns: Wet Tropics Management Authority and Biotropica Australia Pty. Ltd.

[ece370374-bib-0033] Grueber, C. E. , S. Nakagawa , R. J. Laws , and I. G. Jamieson . 2011. “Multimodel Inference in Ecology and Evolution: Challenges and Solutions.” Journal of Evolutionary Biology 24, no. 4: 699–711. 10.1111/j.1420-9101.2010.02210.x. 21272107

[ece370374-bib-0034] Hegarty, E. E. 1991. “Leaf Litter Production by Lianes and Trees in a Sub‐Tropical Australian Rain Forest.” Journal of Tropical Ecology 7, no. 2: 201–214. 10.1017/S0266467400005356.

[ece370374-bib-0035] Heinrich, V. H. A. , R. Dalagnol , H. L. G. Cassol , et al. 2021. “Large Carbon Sink Potential of Secondary Forests in the Brazilian Amazon to Mitigate Climate Change.” Nature Communications 12, no. 1: 1785. 10.1038/s41467-021-22050-1. PMC7979697 33741981

[ece370374-bib-0036] Ingwell, L. L. , S. Joseph Wright , K. K. Becklund , S. P. Hubbell , and S. A. Schnitzer . 2010. “The Impact of Lianas on 10 Years of Tree Growth and Mortality on Barro Colorado Island, Panama.” Journal of Ecology 98, no. 4: 879–887. 10.1111/j.1365-2745.2010.01676.x.

[ece370374-bib-0037] Isnard, S. , and W. K. Silk . 2009. “Moving With Climbing Plants From Charles Darwin's Time Into the 21st Century.” American Journal of Botany 96, no. 7: 1205–1221. 10.3732/ajb.0900045. 21628270

[ece370374-bib-0038] Jiménez‐Castillo, M. , S. K. Wiser , and C. H. Lusk . 2007. “Elevational Parallels of Latitudinal Variation in the Proportion of Lianas in Woody Floras.” Journal of Biogeography 34, no. 1: 163–168. 10.1111/j.1365-2699.2006.01570.x.

[ece370374-bib-0039] Kusumoto, B. , T. Enoki , and Y. Watanabe . 2008. “Community Structure and Topographic Distribution of Lianas in a Watershed on Okinawa, South‐Western Japan.” Journal of Tropical Ecology 24, no. 6: 675–683. 10.1017/S0266467408005452.

[ece370374-bib-0042] Le Saout, S. , M. Hoffmann , Y. Shi , et al. 2013. “Protected Areas and Effective Biodiversity Conservation.” Science 342, no. 6160: 803–805. 10.1126/science.1239268. 24233709

[ece370374-bib-0043] Lertpanich, K. , and Y. Brockelman . 2003. “Lianas and Environmental Factors in the Mo Singto Biodiversity Research Plot, Khao Yai National Park, Thailand.” Natural History Journal of Chulalongkorn University 3, no. 2: 7–17.

[ece370374-bib-0114] Liu, Q. , F. J. Sterck , J. A. Medina‐Vega , et al. 2020. “Soil Nutrients, Canopy Gaps and Topography Affect Liana Distribution in a Tropical Seasonal Rain Forest in Southwestern China.” Journal of Vegetation Science 32, no. 1: e12951. 10.1111/jvs.12951.

[ece370374-bib-0044] Lobos‐Catalán, P. , and M. Jiménez‐Castillo . 2019. “Temperature Shapes Liana Diversity Pattern Along a Latitudinal Gradient in Southern Temperate Rainforest.” Plant Ecology 220, no. 12: 1109–1117. 10.1007/s11258-019-00980-7.

[ece370374-bib-0045] Macía, M. J. , K. Ruokolainen , H. Tuomisto , J. Quisbert , and V. Cala . 2007. “Congruence Between Floristic Patterns of Trees and Lianas in a Southwest Amazonian Rain Forest.” Ecography 30, no. 4: 561–577. 10.1111/j.2007.0906-7590.05124.x.

[ece370374-bib-0046] Mackintosh, E. J. , C. E. Waite , F. E. Putz , S. Brennan , M. Pfeifer , and A. R. Marshall . 2024. “Lianas Associated With Continued Forest Biomass Losses Following Large—Scale Disturbances.” Biotropica 00: e13348. 10.1111/btp.13348.

[ece370374-bib-0047] Malizia, A. , H. R. Grau , and J. W. Lichstein . 2010. “Soil Phosphorus and Disturbance Influence Liana Communities in a Subtropical Montane Forest.” Journal of Vegetation Science 21, no. 3: 551–560. 10.1111/J.1654-1103.2009.01162.X.

[ece370374-bib-0048] Marshall, A. R. , P. J. Platts , R. L. Chazdon , et al. 2020. “Conceptualising the Global Forest Response to Liana Proliferation.” Frontiers in Forests and Global Change 3: 35. 10.3389/ffgc.2020.00035.

[ece370374-bib-0049] Marshall, A. R. , C. E. Waite , M. Pfeifer , et al. 2023. “Fifteen Essential Science Advances Needed for Effective Restoration of the World's Forest Landscapes.” Philosophical Transactions of the Royal Society, B: Biological Sciences 378: 2021065. 10.1098/rstb.2021.0065. PMC9661955 36373922

[ece370374-bib-0050] Marthews, T. , T. Riutta , I. Oliveras Menor , et al. 2014. “Measuring Tropical Forest Carbon Allocation and Cycling: A RAINFOR‐GEM Field Manual for Intensive Census Plots (v3.0).” Manual, Global Ecosystems Monitoring Network 10–39. http://gem.tropicalforests.ox.ac.uk/.

[ece370374-bib-0051] Martínez‐Izquierdo, L. , M. M. García , J. S. Powers , and S. A. Schnitzer . 2016. “Lianas Suppress Seedling Growth and Survival of 14 Tree Species in a Panamanian Tropical Forest.” Ecology 97, no. 1: 215–224. 10.1890/14-2261.1. 27008790

[ece370374-bib-0052] McDowell, N. , C. D. Allen , K. Anderson‐Teixeira , et al. 2018. “Drivers and Mechanisms of Tree Mortality in Moist Tropical Forests.” New Phytologist 219, no. 3: 851–869. 10.1111/nph.15027. 29451313

[ece370374-bib-0053] Metcalfe, D. J. , M. G. Bradford , and A. J. Ford . 2008. “Cyclone Damage to Tropical Rain Forests: Species‐ and Community‐Level Impacts.” Austral Ecology 33, no. 4: 432–441. 10.1111/j.1442-9993.2008.01898.x.

[ece370374-bib-0055] Moran, P. 1950. “Notes on Continuous Stochastic Phenomena.” Biometrika 37: 17–23. 10.2307/2332142. 15420245

[ece370374-bib-0056] Murphy, H. T. , and D. J. Metcalfe . 2016. “The Perfect Storm: Weed Invasion and Intense Storms in Tropical Forests.” Austral Ecology 41, no. 8: 864–874. 10.1111/AEC.12376.

[ece370374-bib-0057] Nagelkerke, N. J. D. 1991. “A Note on a General Definition of the Coefficient of Determination.” Biometrika 78, no. 3: 691–692. 10.1093/biomet/78.3.691.

[ece370374-bib-0112] Nakada, I. , I. Uehara , and H. Mori . 2024. “More Lianas on Larger Host Trees on Steep Slopes in a Secondary Temperate Forest, Japan.” Plant Ecology 225: 519–533. 10.1007/s11258-024-01409-6.

[ece370374-bib-0058] Negrón‐Juárez, R. I. , J. Q. Chambers , G. C. Hurtt , et al. 2014. “Remote Sensing Assessment of Forest Disturbance Across Complex Mountainous Terrain: The Pattern and Severity of Impacts of Tropical Cyclone Yasi on Australian Rainforests.” Remote Sensing 6, no. 6: 5633–5649. 10.3390/RS6065633.

[ece370374-bib-0059] Ngute, A. S. K. , D. S. Schoeman , M. Pfeifer , et al. 2024. “Global Dominance of Lianas Over Trees is Driven by Forest Disturbance, Climate and Topography.” Global Change Biology 30, no. 1: e17140. 10.1111/gcb.17140. 38273497

[ece370374-bib-0060] Odell, E. H. , N. E. Stork , and R. L. Kitching . 2019. “Lianas as a Food Resource for Herbivorous Insects: A Comparison With Trees.” Biological Reviews 94, no. 4: 1416–1429. 10.1111/brv.12508. 30887664

[ece370374-bib-0061] Parolari, A. J. , K. Paul , A. Griffing , et al. 2020. “Liana Abundance and Diversity Increase With Rainfall Seasonality Along a Precipitation Gradient in Panama.” Ecography 43, no. 1: 25–33. 10.1111/ecog.04678.

[ece370374-bib-0062] Pasquini, S. C. , S. J. Wright , L. S. Santiago , and M. Uriarte . 2015. “Lianas Always Outperform Tree Seedlings Regardless of Soil Nutrients: Results From a Long‐Term Fertilization Experiment.” Ecology 96: 1866–1876. 10.1890/14-1660.1. 26378309

[ece370374-bib-0063] Pérez‐Salicrup, D. R. , V. L. Sork , and F. E. Putz . 2001. “Lianas and Trees in a Liana Forest of Amazonian Bolivia.” Biotropica 33, no. 1: 34–47. 10.1646/0006-3606(2001)033[0034:latial]2.0.co;2.

[ece370374-bib-0064] Phillips, O. L. , T. R. Baker , L. Arroyo , et al. 2004. “Pattern and Process in Amazon Tree Turnover, 1976–2001.” Philosophical Transactions of the Royal Society of London. Series B: Biological Sciences 359, no. 1443: 381–407. 10.1098/rstb.2003.1438. 15212092 PMC1693333

[ece370374-bib-0065] Phillips, O. L. , R. Vésquez Martínez , L. Arroyo , et al. 2002. “Increasing Dominance of Large Lianas in Amazonian Forests.” Nature 418, no. 6899: 770–774. 10.1038/nature00926. 12181565

[ece370374-bib-0066] Poggio, L. , E. Simonetti , and A. Gimona . 2018. “Enhancing the WorldClim Data Set for National and Regional Applications.” Science of the Total Environment 625: 1628–1643. 10.1016/j.scitotenv.2017.12.258. 29996459

[ece370374-bib-0067] Poulsen, J. R. , S. E. Koerner , Z. Miao , V. P. Medjibe , L. N. Banak , and L. J. T. White . 2017. “Forest Structure Determines the Abundance and Distribution of Large Lianas in Gabon.” Global Ecology and Biogeography 26, no. 4: 472–485. 10.1111/geb.12554.

[ece370374-bib-0068] Powers, J. S. 2015. “Reciprocal Interactions Between Lianas and Forest Soils.” In Ecology of Lianas, edited by S. A. Schnitzer , F. Bongers , R. J. Burnham , and F. E. Putz , 175–187. Oxford, UK: Wiley‐Blackwell Publishing. 10.1002/9781118392409.ch14.

[ece370374-bib-0069] Putz, F. E. 1984. “The Natural History of Lianas on Barro Colorado Island, Panama.” Ecology 65, no. 6: 1713–1724. 10.2307/1937767.

[ece370374-bib-0070] Putz, F. E. 1990. “Growth Habits and Trellis Requirements of Climbing Palms (*Calamus* Spp.) in North‐Eastern Queensland.” Australian Journal of Botany 38, no. 6: 603–608. 10.1071/BT9900603.

[ece370374-bib-0071] Putz, F. E. 2023. “Climbing Plants Beat Trees to Soil Nutrient Patches.” Current Biology 33, no. 12: R675–R676. 10.1016/j.cub.2023.04.035. 37339592

[ece370374-bib-0072] Putz, F. E. , D. T. Cayetano , E. P. Belair , et al. 2023. “Liana Cutting in Selectively Logged Forests Increases Both Carbon Sequestration and Timber Yields.” Forest Ecology and Management 539: 121038. 10.1016/j.foreco.2023.121038.

[ece370374-bib-0073] Putz, F. E. , and P. Chai . 1987. “Ecological Studies of Lianas in Lambir National Park, Sarawak, Malaysia.” Journal of Ecology 75, no. 2: 523–531. 10.2307/2260431.

[ece370374-bib-0074] R Core Team . 2022. R: A Language and Environment for Statistical Computing. Vienna, Austria: R Foundation for Statistical Computing. https://www.R‐project.org/.

[ece370374-bib-0075] Ramsay, H. A. , and L. M. Leslie . 2008. “The Effects of Complex Terrain on Severe Landfalling Tropical Cyclone Larry (2006) Over Northeast Australia.” Monthly Weather Review 136, no. 11: 4334–4354. 10.1175/2008MWR2429.1.

[ece370374-bib-0076] Reis, S. M. , B. S. Marimon , P. S. Morandi , et al. 2020. “Causes and Consequences of Liana Infestation in Southern Amazonia.” Journal of Ecology 108: 2184–2197. 10.1111/1365-2745.13470.

[ece370374-bib-0077] Roberts, D. W. , and S. V. Cooper . 1989. “Concepts and Techniques of Vegetation Mapping.” In Land Classifications Based onVegetation: Applications for Resource Management, edited by D. Ferguson , P. Morgan , and F. D. Johnson . Ogden, UT: USDA Forestry Service General Technical Report.

[ece370374-bib-0078] Ros‐Tonen, M. A. F. 2000. “The Role of Non‐Timber Forest Products in Sustainable Forest Management.” Holz Als Roh – Und Werkstoff 58, no. 1: 196–201. 10.1007/s001070050413.

[ece370374-bib-0080] Rozali, F. Z. , K. M. Masum , M. S. Mansor , and A. Mansor . 2021. “Rattan Composition and Diversity Assessment in Tropical Rainforests of Peninsular Malaysia for Conservation.” Biodiversity and Conservation 30, no. 11: 2907–2928. 10.1007/s10531-021-02226-3.

[ece370374-bib-0081] Schnitzer, S. A. 2005. “A Mechanistic Explanation for Global Patterns of Liana Abundance and Distribution.” American Naturalist 166, no. 2: 262–276. 10.1086/431250. 16032578

[ece370374-bib-0083] Schnitzer, S. A. , and F. Bongers . 2011. “Increasing Liana Abundance and Biomass in Tropical Forests: Emerging Patterns and Putative Mechanisms.” Ecology Letters 14, no. 4: 397–406. 10.1111/j.1461-0248.2011.01590.x. 21314879

[ece370374-bib-0084] Schnitzer, S. A. , F. Bongers , and Group, M . 2002. “The Ecology of Lianas and Their Role in Forests.” Trends in Ecology & Evolution 17, no. 5: 223–230. 10.1016/S0169-5347(02)02491-6.

[ece370374-bib-0085] Schnitzer, S. A. , and W. P. Carson . 2010. “Lianas Suppress Tree Regeneration and Diversity in Treefall Gaps.” Ecology Letters 13, no. 7: 849–857. 10.1111/j.1461-0248.2010.01480.x. 20482581

[ece370374-bib-0086] Schnitzer, S. A. , J. W. Dalling , and W. P. Carson . 2000. “The Impact of Lianas on Tree Regeneration in Tropical Forest Canopy Gaps: Evidence for an Alternative Pathway of Gap‐Phase Regeneration.” Journal of Ecology 88, no. 4: 655–666. 10.1046/j.1365-2745.2000.00489.x.

[ece370374-bib-0087] Schnitzer, S. A. , D. M. DeFilippis , M. Visser , et al. 2021. “Local Canopy Disturbance as an Explanation for Long‐Term Increases in Liana Abundance.” Ecology Letters 24, no. 12: 2635–2647. 10.1111/ELE.13881. 34536250

[ece370374-bib-0089] Schnitzer, S. A. , S. Estrada‐Villegas , and S. J. Wright . 2020. “The Response of Lianas to 20 Years of Nutrient Addition in a Panamanian Forest.” Ecology 101: e03190. 10.1002/ecy.3190. 32893876

[ece370374-bib-0090] Schnitzer, S. A. , M. E. Kuzee , and F. Bongers . 2005. “Disentangling Above‐ and Below‐Ground Competition Between Lianas and Trees in a Tropical Forest.” Journal of Ecology 93, no. 6: 1115–1125. 10.1111/j.1365-2745.2005.01056.x.

[ece370374-bib-0091] Schnitzer, S. A. , S. A. Mangan , J. W. Dalling , et al. 2012. “Liana Abundance, Diversity, and Distribution on Barro Colorado Island, Panama.” PLoS One 7, no. 12: e52114. 10.1371/journal.pone.0052114. 23284889 PMC3528767

[ece370374-bib-0092] Schnitzer, S. A. , N. L. Michel , J. S. Powers , and W. D. Robinson . 2020. “Lianas Maintain Insectivorous Bird Abundance and Diversity in a Neotropical Forest.” Ecology 101, no. 12: e03176. 10.1002/ECY.3176. 32870500

[ece370374-bib-0093] Schnitzer, S. A. , S. Rutishauser , and S. Aguilar . 2008. “Supplemental Protocol for Liana Censuses.” Forest Ecology and Management 255, no. 3–4: 1044–1049. 10.1016/j.foreco.2007.10.012.

[ece370374-bib-0094] Schnitzer, S. A. , G. M. F. van der Heijden , and J. S. Powers . 2016. “Addressing the Challenges of Including Lianas in Global Vegetation Models.” Proceedings of the National Academy of Sciences of the United States of America 113, no. 1: E5–E6. 10.1073/pnas.1521823113. 26699499 PMC4711824

[ece370374-bib-0116] Siebert, S. F. 1993. “The Abundance and Site Preferences of *Calamus zollingeri* in Two Indonesian National Parks.” Forest Ecology and Management 59, no. 1–2: 105–113.

[ece370374-bib-0095] Smith‐Martin, C. M. , C. L. Bastos , O. R. Lopez , J. S. Powers , and S. A. Schnitzer . 2019. “Effects of Dry‐Season Irrigation on Leaf Physiology and Biomass Allocation in Tropical Lianas and Trees.” Ecology 100, no. 11: e02827. 10.1002/ecy.2827. 31325383

[ece370374-bib-0115] Stiegel, S. , M. Kessler , D. Getto , J. Thonhofer , and S. F. Siebert . 2011. “Elevational Patterns of Species Richness and Density of Rattan Palms (Arecaceae: Calamoideae) in Central Sulawesi, Indonesia.” Biodiversity and Conservation 20, no. 9: 1987–2005. 10.1007/s10531-011-0070-8.

[ece370374-bib-0111] Swaine, M. D. , and J. Grace . 2007. “Lianas May be Favoured by Low Rainfall: Evidence From Ghana.” Plant Ecology 192, no. 2: 271–276. 10.1007/s11258-007-9319-4.

[ece370374-bib-0096] Tang, Y. , R. L. Kitching , and M. Cao . 2012. “Lianas as Structural Parasites: A Re‐Evaluation.” Chinese Science Bulletin 57, no. 4: 307–312. 10.1007/s11434-011-4690-x.

[ece370374-bib-0097] Thonhofer, J. , D. Getto , O. van Straaten , D. Cicuzza , and M. Kessler . 2015. “Influence of Spatial and Environmental Variables on Rattan Palm (Arecaceae) Assemblage Composition in Central Sulawesi, Indonesia.” Plant Ecology 216: 55–66. 10.1007/s11258-014-0416-x.

[ece370374-bib-0098] Tobin, M. F. , A. J. Wright , S. A. Mangan , and S. A. Schnitzer . 2012. “Lianas Have a Greater Competitive Effect Than Trees of Similar Biomass on Tropical Canopy Trees.” Ecosphere 3, no. 2: 873–892. 10.1890/es11-00322.1.

[ece370374-bib-0099] Turton, S. M. 2008. “Landscape‐Scale Impacts of Cyclone Larry on the Forests of Northeast Australia, Including Comparisons With Previous Cyclones Impacting the Region Between 1858 and 2006.” Austral Ecology 33, no. 4: 409–416. 10.1111/j.1442-9993.2008.01896.x.

[ece370374-bib-0100] Turton, S. M. 2019. “Reef‐To‐Ridge Ecological Perspectives of High‐Energy Storm Events in Northeast Australia.” Ecosphere 10, no. 1: e02571. 10.1002/ecs2.2571.

[ece370374-bib-0101] Turton, S. M. , and M. Alamgir . 2015. “Ecological Effects of Strong Winds on Forests.” In Routledge Handbook of Forest Ecology, edited by K. Peh , R. Corlett , and Y. Bergeron , 127–140. Oxford, UK: Routledge. 10.4324/9781315818290.

[ece370374-bib-0102] Tymen, B. , M. Réjou‐Méchain , J. W. Dalling , et al. 2016. “Evidence for Arrested Succession in a Liana‐Infested Amazonian Forest.” Journal of Ecology 104, no. 1: 149–159. 10.1111/1365-2745.12504.

[ece370374-bib-0119] Unwin, G. L. , and P. E. Kriedemann . 1990. “Drought Tolerance and Rainforest Tree Growth on a North Queensland Rainfall Gradient.” Forest Ecology and Management 30, no. 1–4: 113–123. 10.1016/0378-1127(90)90130-4.

[ece370374-bib-0103] van der Heijden, G. M. F. , and O. L. Phillips . 2008. “What Controls Liana Success in Neotropical Forests?” Global Ecology and Biogeography 17, no. 3: 372–383. 10.1111/j.1466-8238.2007.00376.x.

[ece370374-bib-0104] van der Heijden, G. M. F. , and O. L. Phillips . 2009. “Liana Infestation Impacts Tree Growth in a Lowland Tropical Moist Forest.” Biogeosciences Discussions 6, no. 2: 3133–3158. 10.5194/bgd-6-3133-2009.

[ece370374-bib-0105] van der Heijden, G. M. F. , J. S. Powers , and S. A. Schnitzer . 2015. “Lianas Reduce Carbon Accumulation and Storage in Tropical Forests.” Proceedings of the National Academy of Sciences of the United States of America 112, no. 43: 13267–13271. 10.1073/pnas.1504869112. 26460031 PMC4629347

[ece370374-bib-0106] Vogado, N. O. , J. E. Engert , T. L. Linde , M. J. Campbell , W. F. Laurance , and M. J. Liddell . 2022. “Climate Change Affects Reproductive Phenology in Lianas of Australia's Wet Tropics.” Frontiers in Forests and Global Change 5: 787950. 10.3389/ffgc.2022.787950.

[ece370374-bib-0107] Waite, C. E. , G. M. F. van der Heijden , R. Field , et al. 2022. “Landscape‐Scale Drivers of Liana Load Across a Southeast Asian Forest Canopy Differ to the Neotropics.” Journal of Ecology 111: 1–13. 10.1111/1365-2745.14015.

[ece370374-bib-0123] Watanabe, N. M. , and E. Suzuki . 2008. “Species Diversity, Abundance, and Vertical Size Structure of Rattans in Borneo and Java.” Biodiversity and Conservation 17, no. 3: 523–538. 10.1007/s10531-007-9268-1.

[ece370374-bib-0108] Webb, L. J. 1958. “Cyclones as an Ecological Factor in Tropical Lowland Rain‐Forest, North Queensland.” Australian Journal of Botany 6, no. 3: 220–228. 10.1071/BT9580220.

[ece370374-bib-0122] Webb, L. J. 1968. “Environmental Relationships of the Structural Types of Australian Rain Forest Vegetation.” Ecology 49, no. 2: 296–311.

[ece370374-bib-0109] Winter, J. W. , F. C. Bell , and L. I. Pahl . 1987. “Rainforest Clearfelling in Northeastern Australia.” Proceedings of the Royal Society of Queensland 98: 41–57.

[ece370374-bib-0110] Wyka, T. P. , J. Oleksyn , P. Karolewski , and S. A. Schnitzer . 2013. “Phenotypic Correlates of the Lianescent Growth Form: A Review.” Annals of Botany 112: 1667–1681. 10.1093/aob/mct236. 24169592 PMC3838560

[ece370374-bib-0118] Zuur, A. F. , E. N. Ieno , and C. S. Elphick . 2010. “A Protocol for Data Exploration to Avoid Common Statistical Problems.” Methods in Ecology and Evolution 1, no. 1: 3–14. 10.1111/j.2041-210x.2009.00001.x.

